# Analysis of mtDNA control region of an isolated population of Eld’s deer (*Rucervus eldii*) reveals its vulnerability to inbreeding

**DOI:** 10.1080/23802359.2017.1325335

**Published:** 2017-05-13

**Authors:** Sangeeta Angom, Ajit Kumar, Sandeep Kumar Gupta, Syed Ainul Hussain

**Affiliations:** Wildlife Institute of India, Dehradun, Uttarakhand, India

**Keywords:** Eld’s deer, mtDNA, control region, control region, phylogeography, inbreeding depression

## Abstract

The Eld’s deer or brow-antlered deer (*Rucervus eldii*) is one of the most endangered cervids of Southeast Asia. Geographically, it has three distinct subspecies; Sangai or Manipur's brow-antlered deer (*R. e. eldii*), Siamese brow-antlered deer (*R. e. siamensis*) and Thamin or Myanmar’s brow antlered deer (*R. e. thamin*). We examined the genetic diversity of wild and captive populations of *R. e. eldii* and compared its relationship with other subspecies using mtDNA control region gene. During the analysis, only one haplotype was detected in 30 samples of *R. e. eldii.* No genetic variation was observed among the *R. e. eldii* populations. The reduced genetic diversity indicates that the population has passed through the bottleneck effect that might have resulted in the inbreeding depression.

## Introduction

The Eld’s deer (*Rucervus eldii*) is the most localized cervid species found in India occurring as a single isolated population in a 40 km^2^ area of the Keibul Lamjao National Park (KLNP), Manipur (Hussain et al. 2006). Once distributed throughout much of Southeast Asia extending from Manipur to Indochina and southern China, it is now confined in small isolated patches (Gray et al. 2015). Traditional taxonomy divides Eld’s deer into three subspecies; Sangai or Manipur’s brow-antlered deer (*R. e. eldii,* McClelland 1842); Siamese brow-antlered deer (*R. e. siamensis*, Lydekker 1915) and Thamin or Myanmar’s brow antlered deer (*R. e. thamin*, Thomas 1918). A fourth subspecies *R. e. hainanus*, has been recently recognized from the Hainan Island, Southern China (Zhang et al. 2009). Among these, Sangai is the rarest with a localized population of about 100 adult individuals occurring in the southern fringe of the Loktak Lake in Manipur. In fact, the Indian subspecies was considered extinct until a small population of around 14 individuals was rediscovered in the early 1950s (Ranjitsinh 1975). Since then effective conservation measures have re-established the population. In the present study, we assessed levels of genetic diversity of *R. e. eldii* with their sister subspecies using mtDNA control region. Additionally, we compared the wild samples with the captive population, to visualize patterns of differentiation and examine genetic relationships with their sister subspecies.

## Materials and methods

### DNA extraction and sequencing

The tissue samples from dead and decaying carcasses and the faecal pellets of wild and captive populations of Sangai were collected from the KLNP and several zoos in India (Supplementary Table 1). The tissue samples were stored at −20 °C and fresh faecal samples were carefully collected and stored in 70% ethanol at room temperature. DNA was extracted from all the samples using the phenol/chloroform method (Sambrook et al. 1989) and GuHCl-based method (Gupta et al. 2013). PCR amplifications were carried out in 20μl volumes containing 10–40 ng of extracted genomic DNA containing 1 × PCR buffer, 2.0 mM MgCl_2_, 0.2 mM of each dNTP, 3 pmol of each primer, and 0.5 units of AmpliTaq Gold DNA polymerase (Applied Biosystem, Foster City, CA) using primer Cerv.tPro and CervCRH (Balakrishnan et al. 2003). The amplification conditions were as follows: 95 °C for 10 min, followed by 35 cycles at 95 °C for 45 seconds, 55 °C for 45 seconds and 72 °C for 1 min, with a final extension of 72 °C for 10 min. The efficiency and reliability of the PCR reactions were monitored using positive and negative control reactions. The PCR products were electrophoresed on 2% agarose gel and visualized under UV light in the presence of ethidium bromide dye. The amplified PCR product were treated with exonuclease-I and shrimp alkaline phosphatase (USB, Cleveland, OH, USA) for 15 min each at 37 °C and 80 °C, respectively to remove any residual primer. The cleaned PCR products were processed for bi-directional DNA sequencing using the Big Dye Terminator Cycle Sequencing Kit version 3.1 on an ABI 3130 Genetic Analyzer (Applied Biosystems, Foster City, CA). The quality of raw sequences was manually checked using the Sequencher version 4.7 software (Gene codes corporation USA) and editing of data was done with the BioEdit software (Hall 1999).

### Statistical analysis

All the sequences were aligned using the CLUSTAL X program (Thompson et al. 1997) and alignments were checked by visual inspection. Mean pairwise differences between subspecies (Kimura’s 2-parameter) were generated in MEGA 7 (Kumar et al. 2016). DnaSP 5.0 was used to analyze the haplotype (h) and nucleotide (p) diversity (Librado & Rozas 2009). The Bayesian consensus tree was constructed using the Monte Carlo Markov Chain (MCMC) method by BEAST (version 1.7.5; Drummond & Rambaut 2007). Based on the partial sequence of mtDNA control region sequence, phylogenetic tree was generated using the Hasegawa–Kishino–Yano (HKY) model with a constant rate applied across the tree.

## Results

### Geographical distribution of haplotypes

We identified 15 haplotypes from 96 sequences. Of these, 10 were found in 37 samples of *R. e. thamin,* one was found in 30 samples of *R. e. eldii*, three were found in seven samples of *R. e. siamensis*, and one was detected in 22 samples of *R. e. hainanus*. We observed 36 variable nucleotides among 478 base pair long sequence (Table 1), thus accounting for 8.8% viable sites. Four distinct SNPs were detected in *R. e. eldii* on nucleotide (nt) positions (Table 1). The haplotype and nucleotide diversities of each population indicated that the haplotype diversity was high in *R. e. thamin* and *R. e. siamensis*, whereas no genetic diversity was observed in *R. e. hainanus* and captive and wild population of *R. e. eldii*. Based on Kimura’s 2-parameter model, the pairwise genetic distances between haplotypes fall in the range of 0.002–0.049, with the overall average at 0.027. The mean pairwise distance between the populations of *R. e. thamin* and *R. e. eldii* was 0.032 ± 0.008, *R. e. thamin* and *R. e. siamensis* was 0.032 ± 0.007 and *R. e. eldii* and *R. e. siamensis* was 0.039 ± 0.009. These analyses indicated a low pairwise distance between the *R. e. eldii* and *R. e. thamin* suggesting identical mtDNA lineage, whereas high pairwise distance was observed between *R. e. eldii* and *R. e. siamensis.*

**Table 1.** Polymorphic sites within 15 mtDNA control region haplotypes of Eld’s deer and the distribution of haplotypes in each population.

nt position		0	1	1	1	1	1	1	1	2	2	2	2	2	2	2	2	2	2	2	2	2	2	3	3	3	3	3	3	3	3	3	3	3	3	3	4	4	4	4	4	4
		5	0	1	4	6	7	7	9	0	1	3	4	6	6	6	6	7	7	7	8	8	9	0	0	1	1	1	3	3	3	4	6	6	7	9	0	4	4	4	4	6
Pop. (n)	Hap	4	7	9	9	3	5	7	5	9	6	6	4	6	7	8	9	0	1	7	0	8	6	3	6	4	6	7	3	4	5	0	1	8	8	9	9	2	3	5	6	9
RET (4)	H1	C	C	G	T	T	T	A	A	G	C	C	G	T	T	T	C	T	T	T	C	A	C	T	T	A	C	A	A	T	T	T	C	G	T	G	A	G	T	T	C	C
RET (3)	H2	.	.	.	.	C	.	.	.	.	.	.	.	.	.	.	.	.	.	.	.	.	.	.	.	.	.	G	.	.	.	.	.	A	.	.	.	.	.	.	.	.
RET (4)	H3	.	.	.	.	.	.	.	.	.	.	.	.	.	.	.	.	.	.	.	.	.	.	.	.	.	.	G	.	.	.	.	.	A	.	.	.	.	.	.	.	.
RET (10)	H4	.	.	.	.	.	.	.	.	.	.	.	.	.	.	.	.	C	.	.	.	.	.	.	.	G	.	G	.	C	.	.	.	.	.	.	.	.	.	.	T	.
RET (1)	H5	.	.	.	.	.	.	.	.	.	.	.	.	.	.	.	T	.	C	.	.	.	.	.	.	G	.	G	.	C	.	.	.	.	.	.	.	.	.	.	.	.
RET (1)	H6	.	.	.	.	.	.	.	.	.	.	.	.	.	.	.	.	.	.	.	.	.	.	.	.	G	.	G	.	.	.	.	.	.	.	.	.	.	.	.	.	.
RET (7)	H7	.	.	.	.	.	.	.	.	.	.	.	.	.	.	.	.	.	C	.	.	.	.	.	.	G	.	G	.	C	.	.	.	.	.	.	.	.	.	.	.	.
RET (5)	H8	.	.	.	.	.	.	.	.	.	.	T	.	.	.	.	T	.	.	.	.	.	.	.	.	G	.	.	.	.	C	.	.	A	.	A	G	.	.	.	.	.
RET (1)	H9	.	.	.	.	.	.	.	G	.	.	.	.	.	.	.	.	C	C	.	.	.	T	.	.	G	.	G	.	C	.	.	.	.	.	.	.	A	C	.	.	.
RET (1)	H10	.	.	.	.	.	.	.	G	.	.	T	A	.	.	.	T	C	.	.	T	.	T	.	.	G	.	.	.	.	C	.	.	A	.	A	G	A	C	.	.	.
REE (30)	H11	.	.	.	.	.	C	G	G	.	.	.	.	.	.	.	.	C	.	.	.	.	.	.	.	.	T	G	.	.	.	C	T	A	.	.	G	.	.	C	.	T
RES (5)	H12	T	.	C	C	.	.	.	G	.	.	.	.	C	C	C	.	.	C	C	.	.	T	.	.	.	.	.	G	.	.	.	T	A	.	.	G	.	.	C	T	T
RES (1)	H13	T	.	C	.	.	.	.	.	A	T	.	.	C	.	.	.	C	.	C	.	.	.	C	C	.	.	.	G	.	C	.	.	A	.	.	G	.	.	.	T	.
RES (1)	H14	T	T	C	.	.	.	.	.	A	T	.	.	C	.	.	.	C	.	C	.	.	.	C	C	G	.	.	G	.	C	.	.	A	.	.	G	.	.	.	.	.
REH (22)	H15	T	.	C	.	.	.	.	G	.	.	.	.	C	.	.	.	C	.	C	.	G	.	.	.	G	.	.	.	.	.	.	.	A	C	.	G		.	.	T	.

The top three rows of numbers represent the polymorphic nucleotide (nt) positions and dot (.) indicates similarity with the first sequence.

### Phylogenetic status

**Figure 1.**
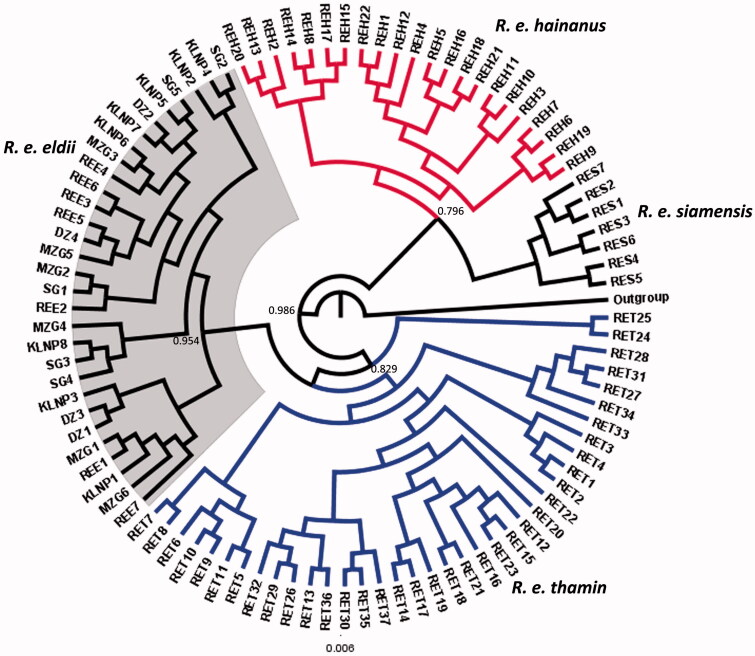
Bayesian (MCMC) consensus tree of 96 Eld’s deer sequences based in the mtDNA control region. Posterior values in percentage are provided at their respective nodes. The *Elaphurus davidianus* (AF291894) was used as an out group. Gray shade represents clade of captive and wild population of Sangai deer (*R. e. eldii*).

All the subspecies of Eld’s deer were explicitly assigned different clade (Figure 1). Large proportion of genetic variations was identified in the *R. e. thamin* clade that indicated an unambiguous population structuring. The wild and captive populations of *R. e. eldii* exhibited single haplotypes and clustered together. The subspecies *R. e. siamensis* and *hainanus* were interspersed, which could reflect some degree of sequence variation within the sampled dataset themselves. It shows that the status of *R. e. siamensis* needs a formal study to examine its accurate taxonomic position. The results further indicated an explicit population structuring within *R. e. thamin* and *R. e. siamensis* population and significant genetic divergence between the subspecies. Diversity measures calculated for the *R. e. thamin* and *R. e. siamensis* showed that both the subspecies have high haplotype and nucleotide diversity, whereas *R. e. eldii* exhibited no variation in the nucleotides.

## Discussion

In the present study, no nucleotide diversity was found in wild and captive populations of *R. e. eldii* which indicate evidence of the bottleneck effect. All individuals of *R. e. eldii* were represented by single haplotype with no genetic variation. It indicated a restricted gene flow among the wild and captive populations of *R. e. eldii*. A possible explanation for the lack of genetic diversity in *R. e. eldii* is the geographical isolation of this subspecies that prevented the gene flow. The fragmented populations adapted the local environmental conditions and utilized limited ecological resources available to them in the floodplains areas compared to its other subspecies may be another cause of low genetic diversity (Hussain et al. 2006). During the last few years, an increasing number of captive populations of *R. e. eldii* were established in different zoos in India from the source stock of Delhi Zoo, further magnifying the effect of inbreeding. Diversity indices were absent in *R. e. eldii* and *R. e. hainanus*, which indicates that the both the populations had gone through a genetic bottleneck in the past. Despite the low genetic distance between *R. e. eldii* and *R. e. thamin* and higher haplotypes in *R. e. thamin* population, the *R. e. eldii* clustered with the *R. e. thamin* in Bayesian consensus tree (Figure 1). The phylogeography of *R. e. eldii, R. e. thamin*, and *R. e. siamensis* indicated clear population structuring and significant genetic divergence between the subspecies. The genetic relationship of Eld’s deer with related cervids suggest that it has a close affinity with the sambar (*Rusa unicolor*) and hog deer (*Axis porcinus*) that is closely related to the chital (*Axis axis*), and were grouped together as monophyly (Angom et al. 2015). The phylogeny of Eld’s deer subspecies revealed monophyly. *R. e. eldii* from Manipur showed a closest relationship with *R. e. thamin* than the *R. e. siamensis*. During the analysis, only one haplotype was detected in wild and captive populations of *R. e. eldii*. The lack of genetic diversity within the *R. e. eldii* indicates that the population is under inbreeding depression.

## Supplementary Material

TMDN_A_1325335_Supplementary_Information.docx
