# Supplementary material for: Analysis of mtDNA control region of an isolated population of Eld’s deer (Rucervus eldii) reveals its vulnerability to inbreeding
Source: Mitochondrial DNA B Resour. 2017 May 13;2(1):277–80. doi: 10.1080/23802359.2017.1325335 (PMC7875480; doi:10.1080/23802359.2017.1325335)
Supplement: TMDN_A_1325335_Supplementary_Information.docx [file TMDN_A_1325335_SM6097.zip › TMDN_A_1325335_SM6097.docx]

**Supplementary** **table 1:** List of the Eld’s deer sub-species and GenBank accession number of mtDNA control region sequences in this study.

| **Sub-species** | **Sample ID** | **N** | **Origin and references** | **Accession Nos.** |
| --- | --- | --- | --- | --- |
| *R. e. eldii* | KLNP1-KLNP8 | 8 | KLNP (Wild) | This study |
|  | MZ1-MZ6 | 6 | Manipur Zoo | This study |
|  | DZ1-DZ4 | 4 | Delhi Zoo | This study |
|  | SG1-SG5 | 5 | Guwahati Zoo | This study |
|  | REE1,REE2 | 2 | Bhaskar & Goyal 2008 | EU870590 ,EU870591 |
|  | REE3-REE7 | 5 | Balakrishnan *et al.* 2003 | AY137117 -AY137121 |
| *R. e.thamin* | RET1-RET25 | 25 | Balakrishnan *et al*. 2003 | AY137087  AY137091 -AY137114 |
|  | RET26-RET37 | 12 | Zhang *et al.* 2009 | FJ851226-FJ851237 |
| *R. e.siamensis* | RES1-RES4 | 4 | Balakrishnan *et al.* 2003 | AY137080-AY137083 |
|  | RES5 | 1 | Randi *et al.* 2001 | AF291892 |
|  | RES6,RES7 | 2 | Zhang *et al.* 2009 | FJ851238, FJ851239 |
| *R. e.hainanus* | REH1-REH3 | 3 | Pang *et al.* 2003 | AF359313-AF359315 |
|  | REH4-REH9 | 6 | Pang *et al.* 2004 | AF359330-AF359335 |
|  | REH10,REH11 | 2 | Balakrishnan *et al.* 2004 | AY137115,AY137116 |
|  | REH12-REH22 | 11 | Zhang *et al*. 2009 | FJ851215-FJ851225 |
